# Pancreatic Lipase Inhibitory Gallotannins from Galla Rhois with Inhibitory Effects on Adipocyte Differentiation in 3T3-L1 Cells

**DOI:** 10.3390/molecules180910629

**Published:** 2013-09-02

**Authors:** O Jun Kwon, Jong-Sup Bae, Ha Yeong Lee, Ju-Young Hwang, Eun-Woo Lee, Hideyuki Ito, Tae Hoon Kim

**Affiliations:** 1 Daegyeong Institute for Regional Program Evaluation, Gyeongsan 712-210, Korea; E-Mail: ojkwon@irpe.or.kr; 2 College of Pharmacy, Research Institute of Pharmaceutical Sciences, Kyungpook National University, Daegu 702-701, Korea; E-Mails: jongsupbae@gmail.com (J.-S.B.); sunnharu@naver.com (H.Y.L.); 3 Department of Food Biotechnology, Andong National University, Andong 760-749, Korea; E-Mail: hjysharp@hanmail.net; 4 Department of Life Science and Biotechnology, Dongeui University, Busan 614-714, Korea; E-Mail: ewlee@deu.ac.kr; 5 Faculty of Health and Welfare Sciences, Okayama Prefectural University, Okayama 719-1197, Japan; E-Mails: hito@fhw.oka-pu.ac.jp; 6 Department of Herbal Medicinal Pharmacology, Daegu Haany University, Gyeongsan 712-715, Korea

**Keywords:** Galla Rhois, gallotannins, pancreatic lipase, protoaphin-*fb*, 3T3-L1, 2-*O*-digalloyl-1,3,4,6-tetra-*O*-galloyl-β-d-glucose

## Abstract

Activity-guided isolation of a methanolic extract of Galla Rhois using pancreatic lipase and 3T3-L1 adipocytes led to the isolation of seven phenolic compounds: protoaphin-*fb* (**1**), 2-*O*-digalloyl-1,3,4,6-tetra-*O*-galloyl-β-d-glucose (**2**), 1,2,3,4,6-penta-*O*-galloyl-β-d-glucose (**3**), 1,2,4,6-tetra-*O*-galloyl-β-d-glucose (**4**), 3-hydroxy-5-methoxy-phenol 1-*O*-β-d-glucoside (**5**), methylgallate (**6**), and gallic acid (**7**). Their structures were established on the basis of NMR and MS spectroscopic data interpretation. All isolates were evaluated for their inhibitory effects on pancreatic lipase, and compounds **1-5** exhibited potent inhibitory effects on this enzyme, with IC_50_ values ranging from 30.6 ± 2.4 to 3.5 ± 0.5 μM. In addition, the highly galloylated compound **2** was also found to induce potent inhibition of adipocyte differentiation in 3T3-L1 cells.

## 1. Introduction

Obesity is the result of an imbalance between energy intake and expenditure and is widely recognized as a major health problem. Obesity has a close association with several pathological disorders, including type II diabetes, hyperlipidemia, hypertension, arteriosclerosis, osteoarthritis, and coronary heart disease [1]. Recent therapeutic approaches to obesity can be categorized according to their distinct mechanisms, such as inhibition of lipases, suppression of energy intake, stimulation of energy expenditure, inhibition of adipocyte differentiation, and control of lipid metabolism [2]. Pancreatic lipase is well known as a key enzyme in absorption of dietary triglyceride. This enzyme, which is secreted from the pancreas, hydrolyzes triglycerides into glycerol and fatty acids [3]. One example is orlistat, a derivative of the naturally occurring potential pancreatic lipase inhibitor produced from *Streptomyces toxytricini*, which is used clinically in treatment of obesity by reducing the energy intake from the diet [4]. Obesity is also characterized at the cellular level by an increase in the number and size of adipocytes differentiated from 3T3-L1 preadipocytes in adipose tissues [5]. Therefore, regulation of fat absorption and lipid accumulation by disturbance of lipase and adipocyte differentiation is considered an important strategy in development of an anti-obesity agent.

Galla Rhois, which is found in the gall of sumacs, has long been recognized as a traditional Korean medicine for treatment of diarrhea, prolonged coughing, and spontaneous perspiration. Several tannic acid and its derivatives recently isolated from this plant were found to exhibit a range of biological activities, including antibacterial [6], antifungal [7] and hepatoprotective [8] effects.

As part of an ongoing investigation for discovery of naturally occurring anti-obesity agents from medicinal plants, a porcine pancreatic lipase assay was used in an initial screening procedure; according to the results, an EtOAc-soluble portion of Galla Rhois extract exhibited significant inhibitory activity, with an IC_50_ value of 6.4 ± 0.7 μg/mL. This paper describes the procedure for isolation and identification of the phenolic constituents **1**–**7** from Galla Rhois, as well as their inhibitory activity against pancreatic lipase and adipocyte differentiation in 3T3-L1 cells.

## 2. Results and Discussion

Successive column chromatographic purification of the EtOAc-soluble fraction of the methanolic extract of Galla Rhois led to isolation and characterization of seven phenolic derivatives **1**–**7**. The isolated known compounds were identified as 2-*O*-digalloyl-1,3,4,6-tetra-*O*-galloyl-β-d-glucose (**2**) [9], 1,2,3,4,6-penta-*O*-galloyl-β-d-glucose (**3**) [10], 1,2,4,6-tetra-*O*-galloyl-β-d-glucose (**4**) [10], 3-hydroxy-5-methoxyphenol 1-*O*-β-d-glucoside (**5**) [11], methylgallate (**6**) [12], and gallic acid (**7**) [12] by comparison of their physicochemical and spectroscopic data (^1^H, ^13^C-NMR, 2D NMR, and MS) with those of authentic samples and reference data.

Compound **1** was obtained as a reddish amorphous powder, [α]^20^_D_ −179.3° (MeOH). Its molecular formula was determined to be C_36_H_38_O_16_ by using negative HRESIMS, which showed a pseudomolecular ion peak at *m/z* 725.2071 [M−H]^−^. The ^1^H-NMR spectrum of **1** in DMSO-*d*_6_ showed the presence of one hydrogen-bonded phenolic proton at *δ*_H_ 13.03 (1H, s, 9′-OH), a set of *meta*-coupled aromatic protons at *δ*_H_ 6.82 (1H, d, *J* = 2.4 Hz, H-8) and 6.14 (1H, d, *J* = 2.4 Hz, H-6), one aromatic proton at *δ*_H_ 6.65 (1H, s, H-8′), three hydroxyl groups at *δ*_H_ 9.40 (1H, s, 10-OH), 9.35 (1H, s, 7-OH), 4.65 (4-OH), six oxymethine protons at *δ*_H_ 5.04 (1H, dd, *J* = 6.6, 3.6 Hz, H-1), 4.77 (1H, dd, *J* = 13.8, 7.2 Hz, H-1′), 3.90 (1H, dd, *J* = 7.8, 1.2 Hz, H-4′), 3.81 (1H, t, *J* = 6.6 Hz, H-3), 3.75 (1H, m, H-3′), and 3.72 (1H, m, H-4). The spectrum also included signals attributable to fourmethyl protons at *δ*_H_ 1.54 (3H, d, *J* = 6.6 Hz, H-11), 1.40 (3H, d, *J* = 7.2 Hz, H-11′), 1.06 (3H, d, *J* = 7.8 Hz, H-12′), and 1.00 (3H, d, *J* = 6.0 Hz, H-12′). In addition to aglycone signals, one characteristic anomeric signal at *δ*_H_ 5.06 (1H, d, *J* = 7.8 Hz, H-1″) and oxygen-bearing protons at *δ*_H_ 3.74–3.25 were observed, indicating the presence of a glucose moiety [13]. These NMR data in combination with 2D NMR experiments performed on **1** suggested that this compound is a dimeric benzo[g]isochromane glucoside [14]. The position of the sugar moiety and linkage of C-5–C-6′ was confirmed from HMBC correlations between H-1’’ and C-9, 4-OH and C-10a, -5, -4a, -4, -3, H-6 and C-9a, 8, -7, -5a, -5, 10-OH and C-10a, -10, -9a, -5a, 4a, H-8′ and C-9′a, 9′, 7′, 6′, and 9′-OH and C-9′a, 9′, 8′, 7′, 5′a [15]. The NOESY correlation between H-8 and H-1″ placed these two *meta*-coupled aromatic protons at H-8 and H-6, respectively. Moreover, analysis of the NOESY spectrum between 12′-CH_3_/H-4′, 1′, 12-CH_3_/H-4, and H-3/11-CH_3_, 4-OH suggested the relative configuration of the remainder of the molecule of **1** to be identical with that of previously reported protoaphin-*fb* [16]. Compound **1** was previously reported as a constituent of *Aphis fabae*, however, its structure elucidation remained unassigned. To the best of our knowledge, this is the first report to elucidate the structure of the fully assigned protoaphin-*fb* derived from a natural source.

**Figure 1:**
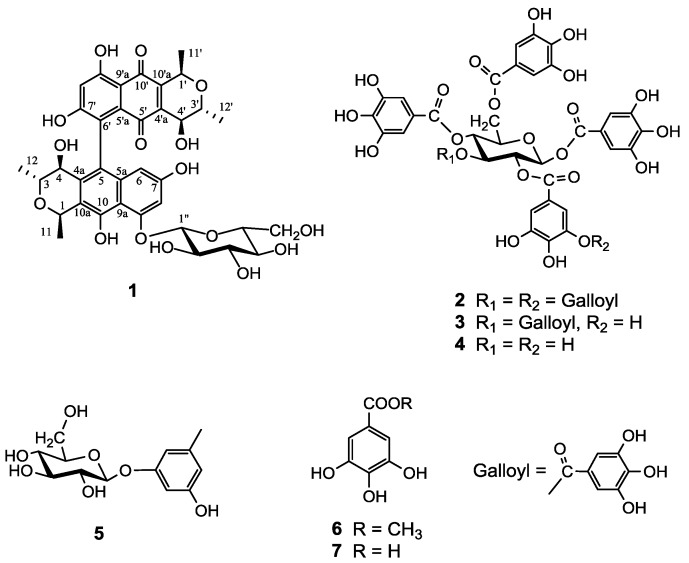
Structures of isolated compounds **1**–**7** from Galla Rhois.

The compounds isolated from Galla Rhois were evaluated for their ability to inhibit pancreatic lipase activity, using orlistat as the positive control (Table 1). In this bioassay system, the known gallotannins 2-*O*-digalloyl-1,3,4,6-tetra-*O*-galloyl-β-d-glucose (**2**), 1,2,3,4,6-penta-*O*-galloyl-β-d-glucose (**3**), and 1,2,4,6-tetra-*O*-galloyl-β-d-glucose (**4**), were found to be the most active, with IC_50_ values ranging from 3.5± 0.5 to 23.2 ± 1.8 *μ*M, while methylgallate (**6**) and gallic acid (**7**) were inactive. In addition, protoaphin-*fb* (**1**) and 3-hydroxy-5-methoxyphenol 1-*O*-β-d-glucoside (**5**) exhibited lower inhibitory effects than the most active compound **2**. Of particular interest, a certain degree of galloylation on the glucose moiety of these active molecules appears to be required for potent inhibitory activity. Furthermore, a 3T3-L1 cell-based adipocyte differentiation assay was performed for evaluation of bioactive compounds **1**, **2**, **3**, and **4** against pancreatic lipase. The non-toxic concentrations of the tested compounds were established in 3T3-L1 preadipocytes using concentrations of 50, 25, and 10 μM. After two days of incubation, an MTT assay was performed for measurement of cell viability; according to the results, no cytotoxic effect was detectable for any of the tested compounds in concentrations up to 50 μM. The inhibitory effects of isolated compounds on differentiation of 3T3-L1 preadipocytes were tested by treatment of the cells with non-toxic concentrations throughout the differentiation period. As shown in Figure 1, Compound **2** induced a significant increase in inhibition of lipid deposition, approximately 64% inhibition, at a concentration of 50 μM. 

**Table 1:** Pancreatic lipase inhibitory activity of compounds **1**–**7**.

Compound	IC_50_ (μM) ^a^
Protoaphin-*fb* (**1**)	30.6 ± 2.4
2-*O*-Digalloyl-1,3,4,6-tetra-*O*-galloyl-β-d-glucose (**2**)	3.5 ± 0.5
1,2,3,4,6-Penta-*O*-galloyl-β-d-glucose (**3**)	15.9 ± 1.0
1,2,4,6-Tetra-*O*-galloyl-β-d-glucose (**4**)	23.2 ± 1.8
3-Hydroxy-5-methoxyphenol 1-*O*-β-d-glucoside (**5**)	78.9 ± 3.7
Methylgallate (**6**)	>300
Gallic acid (**7**)	>300
Orlistat ^b^	0.7 ± 0.2

^a^ IC_50_ values were determined by regression analysis and expressed as mean ± SD of three replicates; ^b^ Used as a positive control.

**Figure 2:**
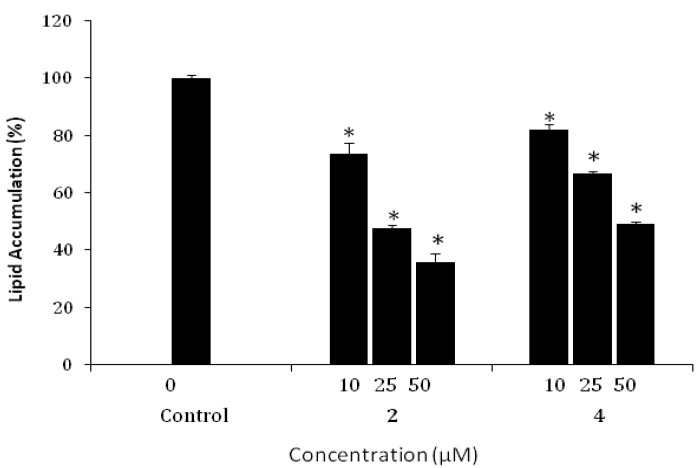
Effect of compounds **2** and **4** on fat accumulation in 3T3-L1. Results are expressed as mean ± SD of three independent experiments, each performed using triplicate wells. * *p* < 0.01 compared with control.

Tannins constitute a large group of plant polyphenols which are believed to have beneficial biological properties, including antioxidant, antiviral, antitumor, and anti-inflammatory activities [17]. Gallotannins are hydrolysable tannins composed of a glucose core esterified to gallic acid residues and widely distributed in dicotyledonous plants. Previously, structure-activity relationships of the representative gallotannins have been investigated extensively for their anticancer [18], antidiabetic [19], and antimicrobial [20] properties and results of these studies suggested that the biological activities of the several gallotannins were influenced primarily by the number and location of the galloyl groups on the sugar moiety. The inhibitory activity against pancreatic lipase of compound **2**, which has six galloyl groups on the glucose moiety, was much stronger than those of gallotannins **3** and **4**, each of which have fewer galloyl residues on the glucose moiety. However, the distinct relationship between structure and activity did not demonstrate that inhibition of adipocyte differentiation in 3T3-L1 cells was dependent on the number and location of galloyl groups attached to the glucose moiety. During the last decade, inhibition of pancreatic lipase and differentiation of adipocytes has been suggested as an important therapeutic approach for prevention and treatment of obesity. In recent years, studies have shown that a number of naturally-occurring gallotannins exhibit anti-obesity activities including suppressing of adipocyte differentiation [21,22]. Based on these facts, 2-*O*-digalloyl-1,3,4,6-tetra-*O*-galloyl-β-d-glucose (**2**) isolated from Galla Rhois showed the most potent inhibitory activity against pancreatic lipase and adipocyte differentiation in 3T3-L1 (Figure 2). Although, several anti-obesity candidates from natural products have been identified [23,24], the potential of highly galloylated gallotannins isolated from Galla Rhois as anti-obesity agents should be noted from the results of this study. Further investigation on the isolation of larger amounts of the potent gallotannins is currently being conducted in order to verify their mode of action.

## 3. Experimental

### 3.1. General

UV spectra were obtained using a Hitachi U-2000 spectrophotometer (Hitachi, Tokyo, Japan). ^1^H- and ^13^C-NMR spectra were measured on a Varian VNS600 instrument (Varian, Palo Alto, CA, USA) operating at 600 and 150 MHz, respectively. The chemical shifts are given in *δ* (ppm) values relative to those of the solvent DMSO-*d*_6_ (*δ*_H_ 2.49; *δ*_C_ 39.7) and acetone-*d*_6_ (*δ*_H_ 2.04; *δ*_C_ 29.8) on a tetramethylsilane (TMS) scale. The standard pulse sequences programmed into the instruments were used for each 2D measurement. The *J*_CH_ value was set at 8 Hz in the HMBC spectra. HRESIMS and ESIMS were obtained on a Micro Mass Auto Spec OA-TOF spectrometer (solvent: 50% MeOH containing 0.1% AcONH_4_; flow rate: 0.02 mL/min; Tokyo, Japan). HPLC analysis was performed on a YMC-Pack ODS A-302 column (4.6 mm i.d. × 150 mm; YMC Co., Kyoto, Japan) and the solvent system consisted of a linear gradient that started with 10% (v/v) MeCN in 0.1% HCOOH/H_2_O (detection: UV 280 nm; flow rate: 1.0 mL/min; temperature: 40 °C), increased to 80% MeCN over 20 min, and then increased to 100% MeCN over 5 min. Column chromatography was performed using Diaion HP-20 (Mitsubishi Kasei Co., Tokyo, Japan), Toyopearl HW-40 (coarse grade; Tosoh Co., Tokyo, Japan), and YMC GEL ODS AQ 120-50S (YMC Co., Kyoto, Japan). Thin-layer chromatography (TLC) was performed on Kieselgel 60 F_254_ plates (0.25 mm layer thickness, Merck, Darmstadt, Germany), and the spots were detected by UV irradiation (254, 365 nm) and by spraying with 10% H_2_SO_4_ reagent.

### 3.2. Plant Material

Galla Rhois was collected in Asan-si, Chungcheongnam-do, Korea in October 2009, and identified by Dr. Tae Hoon Kim. A voucher specimen (KAJ-0077) was deposited at the Natural Product Chemistry Laboratory of Daegu Haany University.

### 3.3. Extraction and Isolation

The dried Galla Rhois (1.2 kg) was ground and extracted exhaustively with MeOH (10 L × 3) at room temperature and the solvent was evaporated *in vacuo*. The combined crude MeOH extract (445.8 g) was suspended in 10% MeOH (1.0 L), and then partitioned in turn with *n*-hexane (2 L × 3), EtOAc (2 L × 3), and *n*-BuOH (2 L × 3) to yield after concentration dried *n*-hexane- (27.6 g), EtOAc- (256.2 g), *n*-BuOH- (37.2 g) and H_2_O-soluble (98.6 g) residues. In a pancreatic lipase inhibition assay, the EtOAc-soluble extract was found to be active, with an IC_50_ value of 6.4 ± 0.7 μg/mL. During chromatographic separation, fractions were monitored by reversed-phase HPLC. A portion (22.0 g) of the EtOAc extract was chromatographed over a Diaion HP-20 column (3.2 cm i.d. × 35 cm) with H_2_O containing increasing amounts of MeOH in a stepwise gradient mode. The H_2_O eluate was subjected to a combination of chromatography over Toyopearl HW-40 column (coarse grade; 3.2 cm i.d. × 57 cm) and YMC GEL ODS AQ 120-50S column (1.1 cm i.d. × 47 cm) with aqueous MeOH to yield pure compounds **2** (5.1 mg, *t*_R_ 11.6 min), **4** (2.0 mg, *t*_R_ 7.3 min), **5** (11.5 mg, *t*_R_ 5.6 min) and **7** (32.0 mg, *t*_R_ 2.5 min). The 40% MeOH eluate from the Diaion HP-20 column (3.2 cm i.d. × 35 cm) was further fractionated by column chromatography on Toyopearl HW-40 (3.2 cm i.d. x 57 cm) and YMC GEL ODS AQ 120-50S (1.1 cm i.d. x 45 cm) with aqueous MeOH to yield pure compound **3** (67.3 mg; *t*_R_ 10.8 min). In a similar fashion, the 60% MeOH eluate was chromatographed over Toyopearl HW-40 column (coarse grade; 3.2 cm i.d. × 57 cm) and YMC GEL ODS AQ 120-50S column (1.1 cm i.d. × 47 cm) with aqueous MeOH to yield pure compounds **1** (24.0 mg, *t*_R_ 13.4 min) and **6** (125.1 mg, *t*_R_ 6.3 min).

*Protoaphin*-*fb* (**1**). Reddish amorphous powder; [α]^20^_D_ –179.3° (*c* 0.1, MeOH); UV (MeOH) λ_max_ (log *ε*): 224 (2.50), 277 (0.80) nm, 314 (0.48); negative ESIMS *m/z* 725 [M-H]^-^, HRESIMS *m/z* 725.2071 [M−H]^−^ (calcd for C_36_H_37_O_16_, 725.2087). ^1^H-NMR (DMSO-*d*_6_): *δ*_H_ 13.03 (1H, s, 9′-OH), 9.40 (1H, s, 10-OH), 9.35 (1H, s, 7-OH), 6.82 (1H, d, *J* = 2.4 Hz, H-8), 6.65 (1H, s, H-8′), 6.14 (1H, d, *J* = 2.4 Hz, H-6), 5.06 (1H, d, *J* = 7.8 Hz, H-1″), 5.04 (1H, dd, *J* = 6.6, 3.6 Hz, H-1), 4.77 (1H, dd, *J* = 13.8, 7.2 Hz, H-1′), 4.65 (1H, s, 4-OH), 3.90 (1H, dd, *J* = 7.8, 1.2 Hz, H-4′), 3.81 (1H, t, *J* = 6.6 Hz, H-3), 3.75 (1H, m, H-3′), 3.74 (1H, m, H-6″), 3.72 (1H, m, H-4), 3.53 (1H, m, H-6″), 3.40 (1H, m, H-5″), 3.38 (1H, m, H-3″), 3.37 (1H, m, H-2″), 3.25 (1H, m, H-4″), 1.54 (3H, d, *J* = 6.6 Hz, H-11), 1.40 (3H, d, *J* = 7.2 Hz, H-11′), 1.06 (3H, d, *J* = 7.8 Hz, H-12′), 1.00 (3H, d, *J* = 6.0 Hz, H-12); ^1^^3^C-NMR (DMSO-*d*_6_): *δ*_c_ 187.5 (C-10′), 182.6 (C-5′), 164.1 (C-7′), 164.0 (C-9′), 155.3 (C-9), 154.5 (C-7), 147.2 (C-10), 144.7 (C-10′a), 142.4 (C-4′a), 134.9 (C-5a), 133.5 (C-4a), 129.6 (C-5′a), 126.1 (C-6′), 123.2 (C-5), 119.1 (C-10a), 109.3 (C-9a), 108.7 (C-9′a), 106.5 (C-8′), 103.4 (C-8), 102.8 (C-1″′), 101.6 (C-6), 77.8 (C-5″’), 76.6 (C-3″′), 73.7 (C-2″′), 69.8 (C-4″′), 69.2 (C-3), 68.7 (C-4), 66.6 (C-1), 66.5 (C-3′), 66.0 (C-1′), 60.7 (C-6″′), 59.1 (C-4′), 19.5 (C-11), 18.7 (C-12′), 18.3 (C-11′), 16.6 (C-12′); ^1^H-NMR (acetone-*d*_6_+D_2_O): *δ*_H_ 7.03 (1H, d, *J* = 2.4 Hz, H-8), 6.73 (1H, s, H-8′), 6.19 (1H, d, *J* = 2.4 Hz, H-6), 5.17 (1H, d, *J* = 7.2 Hz, H-1″′), 5.14 (1H, dd, *J* = 13.2, 6.6 Hz, H-1), 4.82 (1H, dd, *J* = 13.8, 6.6 Hz, H-1′), 4.02 (1H, d, *J* = 1.8 Hz, H-4′), 3.96 (1H, m, H-6″′), 3.95 (1H, m, H-3), 3.94 (1H, m, H-4), 3.85 (1H, ddd, *J* = 7.8, 6.0, 1.8 Hz, H-3′), 3.72 (1H, dd, *J* = 12.0, 6.6 Hz, H-6″′), 3.64 (1H, m, H-2″′), 3.62 (1H, m, H-5″′), 3.61 (1H, m, H-3″′), 3.46 (1H, m, H-4′″), 1.59 (3H, d, *J* = 6.6 Hz, H-11), 1.46 (3H, d, *J* = 6.6 Hz, H-11′), 1.17 (3H, d, *J* = 6.0 Hz, H-12′), 1.03 (3H, d, *J* = 6.0 Hz, H-12′); ^1^^3^C-NMR (acetone-*d*_6_+D_2_O): *δ*_c_ 189.0 (C-10′), 183.4 (C-5′), 166.6 (C-9′), 165.1 (C-7′), 156.3 (C-9), 155.5 (C-7), 149.0 (C-10), 145.7 (C-10′a), 142.9 (C-4′a), 136.3 (C-5a), 134.3 (C-4a), 131.1 (C-5′a), 125.2 (C-6′), 123.2 (C-5), 119.7 (C-10a), 110.7 (C-9a), 110.2 (C-9′a), 107.8 (C-8′), 104.1 (C-8), 103.7 (C-1″′), 102.5 (C-6), 78.3 (C-5″′), 77.7 (C-3″′), 74.4 (C-2″′), 71.0(C-4″′), 70.9 (C-3), 69.7 (C-4), 67.3 (C-3′), 67.2 (C-1), 67.1 (C-1′), 62.3 (C-6″′), 60.6 (C-4′), 20.2 (C-11), 18.3 (C-11′), 18.1 (C-12′), 16.6 (C-12′).

### 3.4. Pancreatic Lipase Activity Assay

A previously reported method [25], with a minor modification, was used for evaluation of the ability of the compounds to inhibit porcine pancreatic lipase. Briefly, an enzyme buffer was prepared by addition of 30 μL (10 units) of a solution of porcine pancreatic lipase (Sigma, St. Louis, MO, USA) in 10 mM morpholinepropanesulphonic acid (MOPS) and 1 mM EDTA (pH 6.8) to 850 μL of Tris buffer (100 mM Tris-HC1 and 5 mM CaCl_2_, pH 7.0). Then, 100 μL of the compounds at the test concentration or orlistat (Roche, Basel, Switzerland) was mixed with 880 μL of the enzyme-buffer, followed by incubation for 15 min at 37 °C, with addition of 20 μL of the substrate solution (10 mM of *p*-nitrophenylbutyrate in dimethylformamide) and the enzymatic reactions were allowed to proceed for 15 min at 37 °C. Pancreatic lipase activity was determined by measuring the hydrolysis of *p*‑nitrophenylbutyrate to *p*-nitrophenol at 405 nm using an ELISA reader (Infinite F200; Tecan Austria GmBH, Grödig, Austria). Inhibition of lipase activity was expressed as the percentage decrease in the optical density (OD) when porcine pancreatic lipase was incubated with the test compounds.

### 3.5. Assay of Adipocyte Differentiation in 3T3-L1 Cells

3T3-L1 mouse embryo fibroblasts were cultured in DMEM supplemented with 10% FBS until reaching confluence. Two days after reaching confluence (day 0), to initiated differentiation, cells were stimulated with differentiation medium containing DMEM with 10% FBS, 0.5 mM 3-isobutyl-1-methylxanthine, 1 μM insulin and 1 μM dexamethasone for two days (day 2). Cells were then maintained in DMEM supplemented with 10% FBS and 2 μM insulin for another six days (day 8), followed by culturing with DMEM with 10% FBS for an additional four days (day 8). All media contained 100 IU/mL penicillin and 100 μg/mL streptomycin. Cells were maintained at 37 °C in a humidified atmosphere of 95% air-5% CO_2_. Throughout the culture period (days 0–8), the cultures were treated with test samples for general experiments [26], with a slight modification. Lipid droplets in cells were stained with Oil Red O. Eight days after induction of differentiation, cells were washed two times with PBS and then fixed with 10% formalin at room temperature for 1 h. After fixation, cells were washed once with 60% isopropyl alcohol and stained with freshly diluted Oil Red O solution (three parts 0.6% Oil Red O in isopropyl alcohol and two parts of water) for 1 h. Cells were then washed twice with water and visualized. For quantitative analysis, Oil Red O staining was dissolved with isopropyl alcohol and optical density was measured at 500 nm using an ELISA plate reader.

## 4. Conclusions

The EtOAc-soluble portion from Galla Rhois extract of Korean origin exhibited potent inhibitory effects on pancreatic lipase, with an IC_50_ value of 6.4 ± 0.7 *μ*g/mL. In the present work, activity-guided isolation of the EtOAc-soluble fraction of Galla Rhois using pancreatic lipase led to the isolation and characterization of seven phenolic metabolites **1**–**7**. The isolation procedure was monitored using a pancreatic lipase assay and the isolated compounds were characterized on the basis of spectrospcopic data interpretation. The known highly galloylated tannins 2-*O*-digalloyl-1,3,4,6-tetra-*O*-galloyl-β-d-glucose (**2**), 1,2,3,4,6-penta-*O*-galloyl-β-d-glucose (**3**), and 1,2,4,6-tetra-*O*-galloyl-β-d-glucose (**4**), were found to be the most active compounds, with IC_50_ values ranging from 3.5 ± 0.5 to 23.2 ± 1.8 μM. Among the isolated potent pancreatic lipase inhibitors, compound **2** was found to display the most potent inhibitory activity for lipid accumulation (64%) at the non-toxic concentration of 50 μM. The bioassay evaluation results observed in present investigation indicate that several gallotannins should be at least in part responsible for the anti-aipogenic activity exhibited by the EtOAc-soluble extract from the methanolic extract of Galla Rhois. 
